# Reply to: Differences in response-scale usage are ubiquitous in cross-country comparisons and a potential driver of elusive relationships

**DOI:** 10.1038/s41598-024-60468-x

**Published:** 2024-05-13

**Authors:** Piotr Sorokowski, Marta Kowal

**Affiliations:** 1 Institute of Psychology, University of Wrocław, Wrocław, Poland; 2 Being Human Lab, Institute of Psychology, University of Wrocław, Wrocław, Poland

**Keywords:** Human behaviour, Cultural evolution

**replying to**: E. Ulitzsch et al.;* Scientific Reports* 10.1038/s41598-024-60465-0 (2024).

In one of our recent studies^1^, we provided evidence of the applicability of the Triangular Theory of Love^2^ across different cultures. Additionally, we observed intriguing but small variations in love experiences across countries. Our key findings suggest that several cultural factors, such as countries’ modernization and individualism levels, as well as environmental factors, such as average annual temperatures might relate to romantic love experiences^3^. Ulitzsch et al.^4^ commented that one of our results concerning a quadratic relationship between romantic love and countries’ Human Development Index (HDI) might result from ‘unaccounted presence of cross-country differences in response styles.’ We agree that the presence of response styles can introduce biases that stem not from genuine differences in the underlying construct but solely from differences in scale usage^5^. However, we caution against prematurely discarding the results of cross-cultural studies that do not explicitly correct for response styles but adopt alternative approaches to assess data quality, such as examining equivalence of invariance.

Likert-type scales are commonly employed in cross-cultural research. Noteworthy, several scholars have emphasized the importance of first establishing the equivalence of invariance before proceeding with any further statistical analyses^6^. Testing equivalence of invariance involves examining whether the scales measure the same latent constructs in all studied countries, making it a golden standard in cross-cultural research^7^. While Liu et al.^8^ demonstrated the adverse impact of response styles on establishing measurement invariance, we did not encounter such issues in our study, as we found evidence supporting the measurement invariance of the TLS-45, the scale we used, across the studied countries. Furthermore, to validate the reliability of our data, we conducted a simple comparison. In our cross-cultural project, participants responded not only to questions regarding love but also their social media usage^9^. If our data is contaminated by the response style bias, regardless of the assessed trait, individuals should exhibit consistent response patterns^10,11^. Contrary to these predictions, the country mean correlation between the TLS-45 and social-media usage was − 0.002 (Kendall rank correlation). Considering that love and social-media usage are rather unrelated, the absence of such a link provides some evidence against a strong response style bias in our data.

In comparison to research on the response style, research on cultural differences and its methodology has a long tradition. Ulitzsch et al. referred to a paper that provided evidence for response styles in diverse cultures^12^. Given that response styles tend to be stable characteristics of respondents across cultures^10,13^, we focused on the findings from Van Herk et al.^12^ and compared the reported response styles in four out of six countries for which we had data. For instance, the authors observed that Greeks tended to provide the most extreme responses, however, in our sample, we observed that the mean level of the TLS-45 in Greece was the second lowest. This is just one example that research on response styles might still need time to develop a strong theoretical basis.

Maybe that is why accommodating the correction for response styles in cross-cultural research using a method suggested by Ulitzsch et al.^4^ is not a common practice. For instance, none of the cross-cultural papers published in the Journal of Cross-Cultural Psychology within 2023 corrected for the response style in a manner suggested by Ulitzsch et al.^4^ Similarly, none of the papers from the largest cross-cultural big data team in psychology (i.e., Psychological Science Accelerator) considered or adjusted for response style in their papers. Nevertheless, we acknowledge that scientific progress calls for scholars to stay abreast of emerging more sophisticated statistical methods that can unravel complex relationships. The current practice might not be the best nor even optimal, and thus, we approached the method proposed by Ulitzsch et al.^4^ with enthusiasm, eager to test it ourselves.

First, we sought to replicate and extend Ulitzsch et al.^4^ models by incorporating missing from their analyses’ components, which were reported in our original paper. Specifically, we aimed to introduce all predictors, including countries’ individualism and average annual temperature, and control for other crucial variables related to love, including participants’ gender and relationship length. Without controlling for these variables, we consider the analyses presented by Ulitzsch et al.^4^ not comparable to our analyses. We also intended to investigate alternative to HDI modernization indexes, such as Modernization Index or Gender Inequality Index, which, as discussed in our paper, tap into the same underlying processes of modernization and exhibit similar patterns of results.

Second, we aimed to treat the three love components as separate factors, in line with the Triangular Theory of Love which we relied on, rather than treating them as a unidimensional factor as done by Ulitzsch et al.^4^ Our two points above are essential because Ulitzsch et al.^4^ applied the response correction to a notably simplified analysis, without controlling for important covariates, originally reported in our paper, which, as we showed, are closely related to the studied phenomenon. Therefore, not including these variables makes Ulitzsch et al.^4^ analyses not comparable to our results.

Third, we wanted to use Ulitzsch et al.^4^ method to recalculate our data by gender and age. Differences between countries tend to be smaller compared to differences within countries^14,15^. This observation also appears to hold true for response styles. Previous studies provided evidence that response style might vary across gender, age, educational background, and personality traits^16,17^. In this context, we wonder if it advisable that the response style correction should be applied to all studies involving, for example, men and women or individuals of different ages? And, what follows, should all the previous analyses involving human subjects and scales–basically, most of the psychological studies to date–be recalculated?

We tried to answer these questions ourselves. In any case, we failed at the very first step when attempting to fit the first Partial Credit Model (PCM) due to the limited computational power of our hardware. Despite running the analysis for 12 h, our computer made only 20% progress. We then attempted to utilize the strongest available computer, but after 11 h, it stopped at 40% progress and crashed. (Specification of the hardware: Strix G15 R9-5900HX/32 GB/512 RTX3060.) Noteworthy, this was only the first model to be estimated, and the three subsequent models that Ulitzsch et al. constructed, and models we suggested would be even more complex. Furthermore, the results of the discussed cross-cultural project included data from over 10,000 participants. In our latest cross-cultural project, we expanded our sample size by tenfold^18,19^. In theory, these analyzes could therefore take several dozen times longer. Consequently, despite our earnest intention, carefully examining the impact of response styles using the method suggested by Ulitzsch et al.^4^ seems almost infeasible. We hope that in the future, these sophisticated methods become more computationally efficient or that the cost of the necessary hardware decreases, rendering their application more accessible. This is particularly important considering that, in theory, the suggested analyses can already be executed using various statistical programs, such as R and Stata.

In summary, we acknowledge that scholars should strive to employ best research practices, including utilizing the most advanced and sophisticated methods. We appreciate Ulitzsch et al.^4^ for raising awareness to an important point in the discussion on cross-cultural methods that warrants further exploration. However, we believe that our study adhered to the most commonly used protocols for cross-cultural research, which involved establishing the equivalence of invariance of the TLS-45 and accounting for the non-independence of participants by introducing multilevel modelling approach^7,20^. The general pattern of results observed in our study concerning the link between modernization and love aligns with findings from other research^21,22^. Importantly, these studies did not employ Likert-type scales but relied on the literary historical analysis^21^ or observations from anthropological field studies^22^, yet, they arrived at similar conclusions, suggesting that modernization might be related to love experiences. Therefore, as also argued by the proponents of considering response style, amending the response style correction might sometimes not clarify but distort our understanding of the studied phenomena^23^, potentially confounding genuine variability with response styles^24^. We anticipate that future investigations will shed more light on our results, potentially even providing contrasting evidence. We would welcome such findings with appreciation, as science is an iterative process in which knowledge accumulates, theories evolve, and new theories emerge. At the same time, we are convinced that certain effects concerning cross-cultural differences are genuine and should not be treated as a result of solely response style biases.
